# Genetic polymorphism and natural selection in the C-terminal 42 kDa region of merozoite surface protein-1 among *Plasmodium vivax* Korean isolates

**DOI:** 10.1186/1475-2875-11-206

**Published:** 2012-06-18

**Authors:** Jung-Mi Kang, Hye-Lim Ju, Yoo-Mi Kang, Dong-Hyun Lee, Sung-Ung Moon, Woon-Mok Sohn, Jae-Won Park, Tong-Soo Kim, Byoung-Kuk Na

**Affiliations:** 1 Department of Parasitology and Institute of Health Sciences, Gyeongsang National University School of Medicine, Jinju 660-751, Korea; 2 Gyeongsang National University Graduate School of Medicine, Jinju, 660-751, Korea; 3 Department of Pathology, College of Medicine, Korea University, Seoul, 136-705, Korea; 4 Department of Microbiology, Graduate School of Medicine, Gachon University of Medicine and Science, Incheon, 406-799, Korea; 5 Department of Parasitology and Inha Research Institute for Medical Sciences, Inha University School of Medicine, Incheon 400-712, Korea

**Keywords:** *Plasmodium vivax*, Merozoite surface protein-1 C-terminal 42 kDa fragment, Genetic diversity, Natural selection, Korea

## Abstract

**Background:**

The carboxy-terminal 42 kDa region of *Plasmodium vivax* merozoite surface protein-1 (PvMSP-1_42_) is a leading candidate antigen for blood stage vaccine development. However, this region has been observed to be highly polymorphic among filed isolates of *P. vivax*. Therefore it is important to analyse the existing diversity of this antigen in the field isolates of *P. vivax*. In this study, the genetic diversity and natural selection in PvMSP-1_42_ among *P. vivax* Korean isolates were analysed.

**Methods:**

A total of 149 *P. vivax-*infected blood samples collected from patients in Korea were used. The region flanking PvMSP-1_42_ was amplified by PCR, cloned into *Escherichia coli*, and then sequenced. The polymorphic characteristic and natural selection of PvMSP-1_42_ were analysed using the DNASTAR, MEGA4 and DnaSP programs.

**Results:**

A total of 11 distinct haplotypes of PvMSP-1_42_ with 40 amino acid changes, as compared to the reference Sal I sequence, were identified in the Korean *P. vivax* isolates. Most of the mutations were concentrated in the 33 kDa fragment (PvMSP-1_33_), but a novel mutation was found in the 19 kDa fragment (PvMSP-1_19_). PvMSP-1_42_ of Korean isolates appeared to be under balancing selection. Recombination may also play a role in the resulting genetic diversity of PvMSP-1_42_.

**Conclusions:**

PvMSP-1_42_ of Korean *P. vivax* isolates displayed allelic polymorphisms caused by mutation, recombination and balancing selection. These results will be useful for understanding the nature of the *P. vivax* population in Korea and for development of a PvMSP-1_42_ based vaccine against *P. vivax*.

## Background

Merozoite surface protein-1 (MSP-1) is a high molecular mass protein abundantly expressed on the surface of the merozoite of malaria parasites and it plays a critical role in the erythrocyte invasion of the parasites [1]. It is synthesized as a large precursor during schizogony and is subsequently processed via proteolytic cleavage into four major polypeptides of approximately 83, 30, 38, and 42 kDa from the N-terminus to C-terminus [1,2]. During the invasion process, the C-terminal 42 kDa fragment (MSP-1_42_) is further processed into 33 kDa (MSP-1_33_) and 19 kDa (MSP-1_19_) fragments, and the latter remains on the merozoite surface and is carried into the invaded erythrocytes, but all the other fragments are released from the merozoite surface [3,4]. Individuals naturally infected with *Plasmodium vivax* acquire humoral immune responses against the C-terminal part of MSP-1, MSP-1_19_ or MSP-1_42_[5-10]. Antibodies that recognize the C-terminal region of *Plasmodium falciparum* MSP-1 inhibit invasion of the merozoites into host erythrocytes *in vitro*[11-14], and immunization of experimental animals with MSP-1_19_ confers protective immunity [15,16]. These findings demonstrate that MSP-1_42_ is a promising candidate antigen for blood stage vaccine development [1,17-19]. However, genetic polymorphisms encoding this region, within and among the *P. vivax* population, are one of the important factors impeding vaccine development.

Vivax malaria had been endemic on the Korean Peninsula for centuries [20], but was eradicated in South Korea by 1979 as a result of intensive efforts led by the World Health Organization and Korean National Malaria Eradication Programme. However, vivax malaria re-emerged in South Korea in 1993 and the outbreak has continued with fluctuating numbers of annual indigenous cases with the total number of cases up to 23,000 [21]. During the early period of the re-emergence, most malaria cases were restricted to military personnel and veterans who served near the Demilitarized Zone (DMZ), and the geographic distribution was limited to the DMZ and adjacent areas where no civilians reside [22]. In spite of the significant decrease in the number of malaria cases among military personnel since the re-emergence, mainly resulting from aggressive chemoprophylaxis, the number of malaria cases in the civilian population has increased and the geographic distribution is expanding into southward cities and counties nearby the DMZ, a pattern indicating the establishment of local transmission in South Korea [21,23].

Although genetic polymorphisms in the central repeat region of MSP-1 in Korean *P. vivax* isolates has been previously analysed [24-26], little information is available regarding the genetic polymorphism of MSP-1_42_ among Korean *P. vivax* population. In this study, the genetic polymorphisms and natural selection in MSP-1_42_ among *P. vivax* Korean isolates were analysed to gain in-depth understanding of the nature of Korean *P. vivax* population. The results suggested that a significant level of genetic diversity exists in the MSP-1_42_, particularly in MSP-1_33_, among Korean *P. vivax* isolates and the region is undergoing a natural selection process.

## Methods

### Blood samples

A total of 149 blood samples were collected from Korean patients infected with *P. vivax* in Korea between 1999 and 2010. *Plasmodium vivax* infection was identified by microscopic examination of thin and thick blood smears and confirmed by polymerase chain reaction (PCR) [27]. All the patients have a febrile illness and have not been abroad at least in recent two years when their blood samples were collected. About 5 ml of blood was collected from each individual. The blood was separated into packed cells and plasma and then stored at −80°C until use. Blood collections performed for this study were conducted following informed consent of the patients and adhering to the institutional ethical guidelines reviewed and approved by either the Ethics committee of Gachon University of Medicine and Science or Inha University School of Medicine.

### Genomic DNA extraction and amplification of PvMSP-1_42_

Genomic DNA was extracted from 200 μl of blood sample using a QIAamp Blood Kit (Qiagen, Valencia, CA, USA). Amplification of PvMSP-1_42_ was performed using two rounds of PCR with primers described previously [28]. The first round PCR primers were 5'-ACGTAGCAGCAAAAGCGCAG-3' and 5'-GCAACATGAGCAACAAGAAGG-3' and the primers for nested PCR were 5'-ACTACGCCGAGGACTACGAC-3' and 5'-AGGACAAGCTTAGGAAGCTGG-3'. The amplification reaction for each round of PCR was performed using the following thermal cycling conditions: 94°C for 5 min, 30 cycles at 94°C for 1 min, 50°C for 1 min, and 72°C for 1.5 min, and followed by a final extension at 72°C for 10 min. Ex Taq DNA polymerase (Takara, Otsu, Japan) was used in all PCR reactions in order to eliminate any possible nucleotide misincorporation. The PCR product was analysed on a 1.2% agarose gel, purified from the gel, and then ligated into the T&A cloning vector (Real Biotech Cooperation, Banqiaa City, Taiwan). Each ligation mixture was transformed into *Escherichia coli* DH5α competent cells and positive clones with the appropriate insert were selected. The nucleotide sequences of the cloned insert were analysed by automatic DNA sequencing. In order to verify the sequences, at least two clones from each isolate were sequenced in both directions. Some isolates underwent three-fold sequence coverage to confirm the existence of rare polymorphisms. The nucleotide sequences reported in this study have been deposited in the GenBank database under the accession numbers JQ446312-JQ446322.

### Sequence and phylogenetic analysis

Nucleotide and deduced amino acid sequences of PvMSP-1_42_ were analysed using EditSeq and SeqMan in the DNASTAR package (DNASTAR, Madison, WI, USA). The phylogeny tree was constructed using the neighbour-joining method in MEGA4 [29]. Bootstrap proportions were used to assess the robustness of the tree with 1,000 bootstrap replications.

### DNA sequence polymorphism analysis

DNA sequence polymorphism analysis was performed on the 149 PvMSP-1_42_ sequences. The number of segregating sites (S), haplotypes (H), haplotype diversity (Hd), nucleotide diversity (π), and the average number of pair-wise nucleotide differences within the population (*K*) were estimated using DnaSP ver. 5.10.00 [30]. The π was calculated on a sliding window of 100 bases with a step size of 25 bp to estimate the stepwise diversity across PvMSP-1_42_. The rates of synonymous (dS) and non-synonymous (dN) substitutions were estimated and compared by the Z-test (P < 0.05) in MEGA4 program [29] using the Nei and Gojobori’s method [31] with the Jukes and Cantor correction. Tajima’s D test [32] was performed with DnaSP ver. 5.10.00 to evaluate the neutral theory of evolution. Fu and Li’s D and F statistics [33] were also analysed using DnaSP ver. 5.10.00 [30].

### Recombination parameters and linkage disequilibrium

The recombination parameter (R), which included the effective population size and probability of recombination between adjacent nucleotides per generation, and the minimum number of recombination events (Rm) were measured using DnaSP ver. 5.10.00 [30]. Linkage disequilibrium (LD) between different polymorphic sites was computed in terms of the R^2^ index.

## Results

### Genetic polymorphisms and amino acid changes

**Figure 1:**
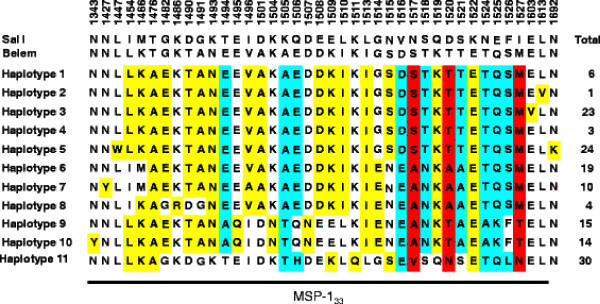
**Sequence polymorphism of PvMSP-1 **_**42**_** in Korean *Plasmodium vivax *isolates.** Sequence analysis revealed that a total of 11 distinct haplotypes of PvMSP-1_42_ were identified in 149 *P. vivax* Korean isolates. Polymorphic amino acids compared to the reference sequence, Sal I (AF435593), are listed for each haplotype. The dimorphic, trimorphic and tetramorphic amino acid changes are marked in yellow, blue and red, respectively. The total numbers of sequences for each haplotype are listed in the right panel.

**Figure 2:**
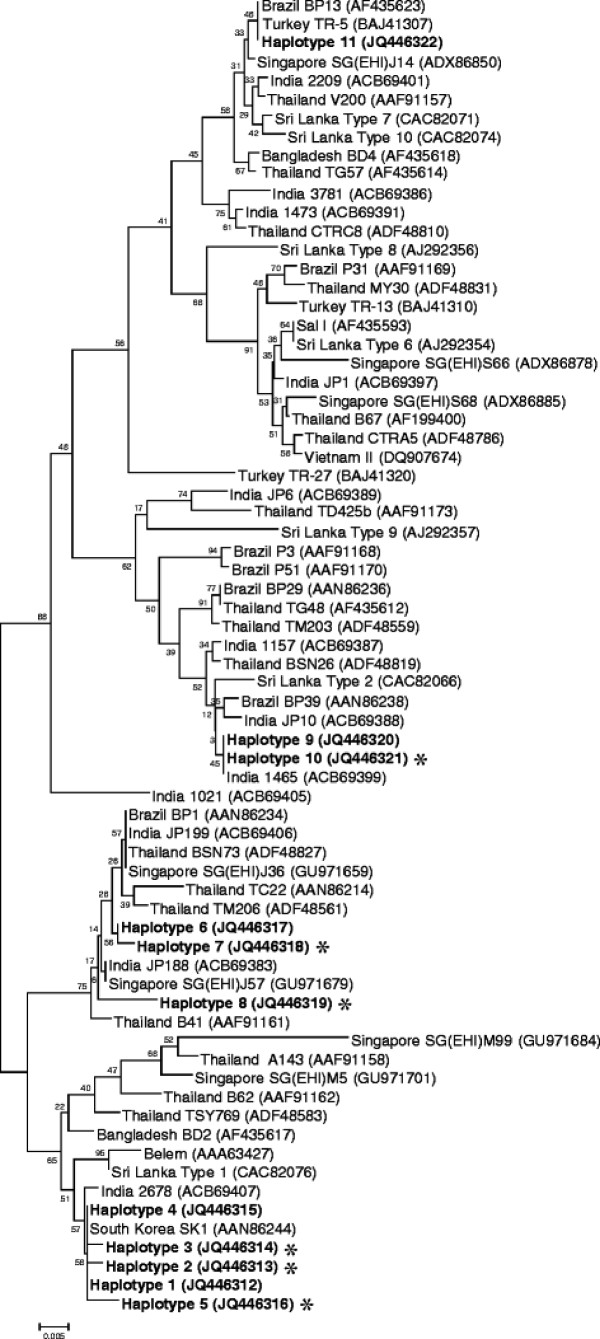
**Phylogenetic analysis.** The phylogenetic tree for the 11 haplotypes of PvMSP-1_42_ was constructed using a neighbour-joining method with the MEGA4 program. Numbers on the branches indicate bootstrap proportions (1,000 replicates). The new haplotypes are marked with asterisks.

**Figure 3:**
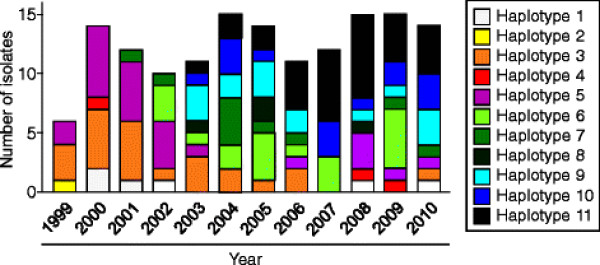
**Annual distribution of PvMSP-1**_**42**_** haplotypes during the study period.** The 149 PvMSP-1_42_ sequences from Korean isolates were analysed by year of collection. Only Belem type haplotypes of PvMSP-1_42_ were identified during 1999–2000. Recombinant types began to be identified in 2001, and both Belem-type and recombinant type haplotypes were identified thereafter.

The region corresponding to PvMSP-1_42_ was amplified from the 149 *P. vivax* Korean isolates. Nucleotide sequence analysis of the 149 PvMSP-1_42_ sequences revealed that there was no size variation between the sequences but they showed polymorphic characteristics. A sequence analysis of the 149 PvMSP-1_42_ sequences resulted in their classification into 11 different haplotypes (haplotypes 1–11) with amino acid changes at 40 positions as compared to the reference Sal I sequence (Figure 1). Of the 40 amino acid changes, three were tetramorphic (N1517S/A/V, D1520T/A/N and I1527M/T/N), nine were trimorphic (T1494E/A, K1505A/T, Q1506E/H, V1516D/E, S1518T/N, S1521T/A, N1524T/A, E1525Q/K and F1526S/L), and the others were dimorphic (Figure 1). Most of the amino acid substitutions were found in the PvMSP-1_33_ region and only one dimorphic substitution (N1692K) was identified in PvMSP-1_19_. Interestingly, seven amino acid changes (N1343Y, N1427Y, L1447W, K1486R, E1603V, L1613V, and N1692K) were unique and had not been identified previously, and represented novel haplotypes. Sequence analysis of the 11 haplotypes of PvMSP-1_42_ based on the hypervariable region also revealed that haplotypes 1–5 were essentially similar to the Belem type. However, the others (haplotypes 6–11) were recombinant forms between Sal I and Belem, which had at least one possible recombination site in their sequences. Phylogenetic analysis revealed that the Korean PvMSP-1_42_ haplotypes were clustered into five clades, a Belem-type and four scattered clades (Figure 2). Upon analysis of the distribution of each MSP-1_42_ haplotype in each year, an interesting finding was observed. In the isolates collected in 1999–2000, only the Belem type haplotypes of PvMSP-1_42_ were identified. However, a recombinant type haplotype (haplotype 7) was first identified among the isolates collected in 2001, and both Belem and recombinant haplotypes were identified thereafter with a prevalence of recombinant haplotypes (Figure 3).

### Nucleotide diversity and natural selection of PvMSP-1_42_

**Figure 4:**
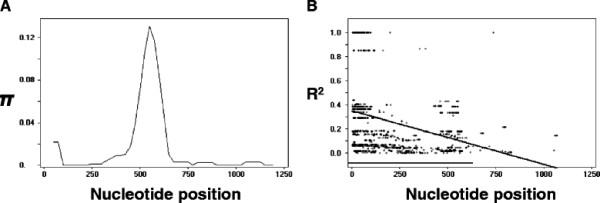
**Natural selection and recombination event of PvMSP-1**_**42**_**.** (**A**) Sliding window plot of nucleotide diversity per site (π) comparing the level of genetic diversity at PvMSP-1_42_. The π values were calculated using DnaSP with a window length of 100 bp and step size of 25 bp. (**B**) The linkage disequilibrium (LD) plot showing non-random association between nucleotide variants in the 149 Korean *P. vivax* isolates at different polymorphic sites. The R^2^ values were plotted against nucleotide distance using a two-tailed Fisher’s exact test for significance.

DNA sequence analyses were performed to determine the nucleotide diversity and genetic differentiation at PvMSP-1_42_ among the Korean *P. vivax* isolates. The average number of pair-wise nucleotide differences (*K*) for the 1,234 bp PvMSP-1_42_ region was 19.570 (Table 1). The overall haplotype diversity (Hd) and nucleotide diversity (π) for all 149 sequences was 0.876 ± 0.009 and 0.01586 ± 0.00047, respectively (Table 1). Analysis of the genetic diversity of the PvMSP-1_33_ and PvMSP-1_19_ fragments revealed that the PvMSP-1_19_ fragment is more highly conserved than the PvMSP-1_33_ fragment, indicating that most of the nucleotide diversity was concentrated in PvMSP-1_33_. The overall haplotype diversity (Hd) and nucleotide diversity (π) for PvMSP-1_33_ was 0.873 ± 0.009 and 0.02051 ± 0.00063, respectively. To examine whether or not natural selection contributed to the diversity observed in PvMSP-1_42_ within the Korean *P. vivax* population, the average difference of dN-dS for all PvMSP-1_42_ sequences was analysed. The estimated dN-dS was 0.0067, indicating that positive natural selection may be occurring in the PvMSP-1_42_ of Korean isolates (Table 1). PvMSP-1_33_ and PvMSP-1_19_ also showed positive dN-dS values of 0.0085 and 0.0016, respectively. In order to more closely explore natural selection in the PvMSP-1_42_, Tajima’s D test was applied and the value was estimated to be 3.0268 (P < 0.01), indicating that PvMSP-1_42_ is under positive selection pressure (Table 1). The Tajima’s D values for PvMSP-1_33_ and PvMSP-1_19_ also showed positive values of 3.0556 (P < 0.01) and 0.5504 (P > 0.1), respectively. The Fu and Li’s D and F values for PvMSP-1_42_ were 2.0839 (P < 0.02) and 2.9904 (P < 0.02), respectively. Analysis of the sliding window plot (window length 100 bp, step size 25 bp) using the DnaSP package revealed that π ranged from 0 to 0.1301 and supported our observations that most of the variations were concentrated between nucleotide positions 400–675, corresponding to the middle region of PvMSP-1_42_ (Figure 4A).

**Table 1:** DNA sequence polymorphisms in PvMSP-1 C-terminal fragment among Korean isolates

**MSP-1 Fragment**	**Segregating sites (S)**	**Singleton variable sites**	**Parsimony informative sites**	**Total no. of mutations**	***K***	**H**	**Hd ± SD**	**Π ± SD**	**dN-dS**	**Tajima’s D**	**Fu and Li’s D**	**Fu and Li’s F**
MSP-1_19_	1	0	1	1	0.272	2	0.272 ± 0.041	0.00093 ± 0.00014	0.0016	0.5504 (P > 0.1)	0.4693 (P > 0.1)	0.5786 (P > 0.1)
MSP-1_33_	51	1	50	54	19.317	11	0.873 ± 0.009	0.02051 ± 0.00063	0.0085	3.0556 (P < 0.01)	2.0713 (P < 0.02)	2.9959 (P < 0.02)
MSP-1_42_	52	1	51	55	19.570	11	0.876 ± 0.009	0.01586 ± 0.00047	0.0067	3.0268 (P < 0.01)	2.0839 (P < 0.02)	2.9904 (P < 0.02)

*K*, average number of pairwise nucleotide differences; H, number of haplotypes; Hd, haplotype diversity; π, observed average pairwise nucleotide diversity; *K*n, rate of non-synonymous mutations; *K*s, rate of synonymous mutations.

### Recombination

For PvMSP-1_42_, the minimum number of recombination events between adjacent polymorphic sites (Rm) was five, whereas the R between adjacent sites (Ra) and per gene (Rb) was 0.0065 and 8.0, respectively. Similar results were obtained when PvMSP-1_33_ was analysed (Rm: 5, Ra: 0.0080, and Rb: 7.5). These high recombination parameter values suggested that meiotic recombination may be occurring between sites, resulting in genetic diversity of the PvMSP-1_42_. The LD index, R^2^, also declined across the analysed region, suggesting that intragenic recombination may also be a possible factor contributing to the diversity observed in PvMSP-1_42_ (Figure 4B).

## Discussion

A blood stage malaria vaccine ideally aims to prevent or considerably reduce blood stage parasitaemia either by inhibiting merozoite invasion into erythrocytes or by targeted destruction of parasitized erythrocytes [19,34]. Following this approach, several merozoite surface proteins (MSPs) have been considered promising candidate antigens for malaria vaccine development due to their accessibility by antibodies and their essential roles in erythrocyte invasion [19]. However, the genetic diversity of the MSPs identified within and among global isolates has resulted in a major obstacle hampering the development of an effective malaria vaccine. Of the MSPs, MSP-1_42_ is the most outstanding vaccine candidate antigen, which is currently at an advanced stage of clinical evaluation [34-37]. But, its polymorphic nature suggests that routine changes to the vaccine and continuous surveillance of the antigen diversity in field isolates would be required.

In this study, the genetic diversity and natural selection on PvMSP-1_42_ in the 149 *P. vivax* Korean isolates were analysed. The 149 sequences were classified into 11 distinct haplotypes with amino acid changes at 40 positions as compared to the Sal I sequence. Most of the amino acid substitutions were concentrated in the PvMSP-1_33_ fragment and only a dimorphic change (N1692K) was found in PvMSP-1_19_. It is known that PvMSP-1_19_ is highly conserved, as observed in field isolates obtained from different geographic regions, and only one amino acid change (K1709E) has been reported thus far [38-41]. The amino acid change was not observed in any of the Korean isolates, but the emergence of a new amino acid change in PvMSP-1_19_ in Korean isolates suggest that PvMSP-1_19_ could contribute to the diversity of PvMSP-1_42_. Of the 39 amino acid changes found in PvMSP-1_33_ of Korean isolates, six (N1343Y, N1427Y, L1447W, K1486R, E1603V, and L1613V) were unique and had not been reported previously. These unique changes resulted in the generation of six novel haplotypes that had not been reported so far. The sequence and phylogenetic analyses revealed that none of the Korean haplotypes were identical to either the Sal I or Belem sequences, but haplotypes 1–5 were essentially Belem types, and the others were recombinant types between Belem and Sal I, in which at least one recombination may occur at the hypervariable region of PvMSP-1_33_. Recently, PvMSP-1_42_ was differentiated into 12 distinctive groups (group 1–12) based on sequence differences observed in hypervariable region, but there was no evidence of geographic clustering of global isolates [42]. Phylogenetic analysis of Korean PvMSP-1_42_ haplotypes suggested they were clustered into five distinct clades with the majority belonging to the Belem type, but no clear geographic relationship was also identified. Interestingly, the isolates collected in 1999–2000 showed only limited haplotypes which were closely related to the Belem type. However, a recombinant haloptype was first observed among isolates collected in 2001. Both Belem and recombinant types of the PvMSP-1_42_ haplotypes were identified thereafter, with a prevalence of the recombinant types. These results coincided with several previous studies based on the genetic diversity of several major antigens including circumsporozoite protein, MSP-1, and MSP-3α, as well as microsatellite loci, suggesting that the Korean *P. vivax* isolates had been genetically homologous until 2000, but the genetic diversity was rapidly disseminated thereafter [26,43]. It is currently unclear why Korean *P. vivax* isolates showed such diverse genetic profiles and this issue should be elucidated. PvMSP-1_19_ was found to be more highly conserved than PvMSP-1_33_ in the Korean isolates, but the nucleotide diversity (π) of PvMSP-1_19_ was considerably higher than those found in previous studies [28,42,44]. This was due to a non-synonymous substitution (N1692K) in PvMSP-1_19_ of the Korean isolates. Meanwhile, the π values for PvMSP-1_42_ and PvMSP-1_33_ found in the Korean isolates were lower than those observed in other global isolates [28,42,44]. This suggests that PvMSP-1_42_ of Korean isolates showed limited genetic diversity as compared to isolates from other endemic regions including India and Sri Lanka.

The rate of non-synonymous and synonymous mutations (dN-dS) is widely used as an indicator of the action of natural selection in gene sequences. An excess of dN relative to dS is a clear signal of positive selection, whereas a lack of dN relative to dS suggests a negative or purifying selection imposed by functional constraints [31,45]. The positive value of dN-dS (0.0067) observed in the 149 Korean PvMSP-1_42_ sequences suggested that PvMSP-1_42_ in the Korean *P. vivax* isolates is under the influence of positive natural selection. The observation that PvMSP-1_33_ had a higher dN-dS than PvMSP-1_42_ also suggested that PvMSP-1_33_ is under stronger positive natural selection pressure than the entire PvMSP-1_42_, and this finding was comparable to observations found in *P. vivax* isolates from different areas [28,42]. The positive values of Tajima’s D (3.0268, P < 0.01) and Fu and Li’s D (2.0839, P < 0.02) and F (2.9904 < 0.02) statistics indicated that the PvMSP-1_42_ alleles occurred at more intermediate frequencies than expected and that few alleles were rare or near fixation, which is consistent with the action of the balancing selection that maintains allelic variation in a population. These results collectively suggested that strong balancing selection, presumably by host immune pressure [28,45,46], occurred at PvMSP-1_42_ in the Korean isolates, and the host immune responses likely played a role in generation and maintenance of the MSP-1_42_ polymorphism.

The diversity of plasmodial antigens is also likely to be generated by genetic recombination during the sexual stage of the parasites in the mosquito [45,46]. The results obtained in this study indicated that recombination events occurred within the PvMSP-1_42_ sequences in Korean isolates. This was supported by the observation of decline of LD index R^2^ with increasing nucleotide distance coupled with a high level of haplotype diversity (Hd = 0.876 ± 0.009). Indeed, all recombinant types of the Korean PvMSP-1_42_ haplotypes had putative recombination sites that concentrated in PvMSP-1_33_ rather than being evenly distributed across the entire PvMSP-1_42_, which consistent with previous reports [28,41,42]. Considering the first appearance of the recombinant haplotype PvMSP-1_42_ in 2001 and the subsequent prevalence of recombinant types from 2003 to recent years, new PvMSP-1_42_ haplotypes are actively being generated in Korean isolates by recombination events in recent years even though the country with low malaria transmission rate.

## Conclusion

This study provided the first in-depth analysis of the genetic diversity and natural selection of PvMSP-1_42_ in Korean *P. vivax* isolates. PvMSP-1_42_ showed polymorphic characteristics that resulted in 11 distinct haplotypes of the Belem or recombinant types. Most of the observed amino acid changes were identified in PvMSP-1_33_, but a novel amino acid change that had not been reported in global isolates was identified in PvMSP-1_19_. Considering the low transmission rate and unstable malaria conditions in Korea, both interallelic and intragenic recombinations are likely to play roles in the generation and maintenance of the diversity of PvMSP-1_42_. Furthermore, balancing selection in response to host immune responses may also contribute to the diversity of PvMSP-1_42_ in Korean isolates. Theses results will be helpful in understanding the nationwide parasite heterogeneity and the implementation of malarial control programmes in Korea, as well as for the development of a PvMSP-1 based vaccine against *P. vivax*.

## Competing interests

The authors declare that they have no competing interests.

## Authors’ contributions

JMK, HLJ, YMK, and DHL performed all the experiments and analysed the sequence data. SUM performed sequence and phylogenetic analyses. JWP and TSK collected the blood samples. BKN and TSK designed the study and supervised the study process. BKN wrote the paper. TSK and WMS assisted in writing and editing the manuscript. All authors read and approved the final manuscript.
